# Method for Preventing the Projection of the Bones in Fracture of the Leg

**Published:** 1847-06

**Authors:** 


*Method for preventing the projection of the Bones in Fracture of the Leg.*—The leg is to be placed in the bent position on the outside, with a common side splint placed above and below, slightly hollowed out to fit the leg. In addition to these, two straight splints are used, padded on one side,—one of sufficient length to extend from the patella to the upper part of the lower third of the leg, the other long enough to reach from the hollow of the knee to beyond the heel. If the straps be now passed round the leg, including the shorter of the two straight splints on the front, and the longer splint on the back of the leg, along with the two bottom splints on the upper and under side, the tibia and fibula above the fracture will be pushed backwards, whilst the foot with the part below the fracture is pressed forwards. In this manner the tendency of the tibia to pass forwards, after simple dislocation or fracture near the ankle, is effectually presented.—*Ibid, from Ormerod's Clinical Observations.*

